# 2595. Burden of Lower Respiratory Tract Infection in United States of America and its Trend from 1990-2019: A Benchmarking Analysis from the Global Burden of Disease Study

**DOI:** 10.1093/ofid/ofad500.2210

**Published:** 2023-11-27

**Authors:** Deepti Raj, Sahithi Rao Mallyala, Avinash Chirumamilla, Jay Gajjar, Sneh Patel, Anushka Dekhne, Hardik Dineshbhai Desai

**Affiliations:** K.S. Hegde Medical Academy, Manglore, Karnataka, India; Dr. NTR University of Health Sciences, Vijaywada, Andhra Pradesh, India; Yenepoya Medical College, Manglore, Karnataka, India; Smt. NHL Municipal Medical College, Ahmedabad, Gujarat, India; GMERS Medical College, Gandhinagar, Gandhinagar, Gujarat, India; American University of Antigua, St Johns, Antigua, Osbourn, Saint John, Antigua and Barbuda; Gujarat Adani Institute of Medical Sciences, Affiliated K.S.K.V University, Ahmedabad, Gujarat, India

## Abstract

**Background:**

Lower respiratory infections (LRIs) cause substantial mortality and morbidity in the United states of America (USA) accounting for 2.78% of all causes of deaths. However, there is a large statewide variation within the United States with regard to LRIs burden. Comparable and consistent state-level measures of total LRIs burden have not been produced previously.

**Methods:**

We used Global Burden of Disease Methodology to estimate the burden of LRIs by Age-groups, year, sex, location across the USA.

**Results:**

In 2019, there were 299,564(95% UI 281,732–317,390) prevalent cases of LRIs in the USA, with an age-standardized rate (ASR) of 85.2 (95% UI 78.8-91.9) per 100,000 population. The annual percentage change of total number incidence increased by 14% followed by deaths increased 13% from 1990-2019. Furthermore, Mississippi [20.49 (95% UI 17.4–23.9)] per 100,000 and Tennessee [19.1 (95% UI 16–22.5)]per 100,000 had the highest age-standardized mortality rate (ASMR) in 2019. In 2019, the incidence and DALY were highest in the 60-64 age group,75-79 age group respectively in both females and males.

Age-Standardized Incidence, Mortality and DALYs Lower Respiratory Tract Infection in United States, 2019

**Figure:**
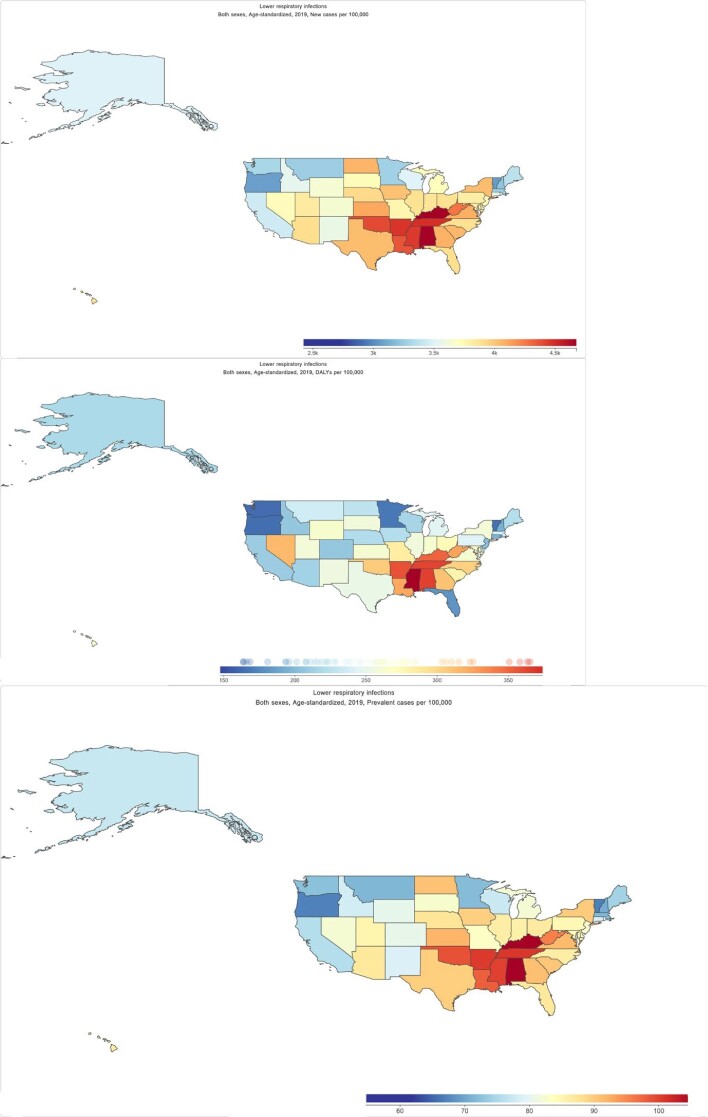

Trend of Lower Respiratory Tract Infection in USA from 1990-2019

**Figure:**
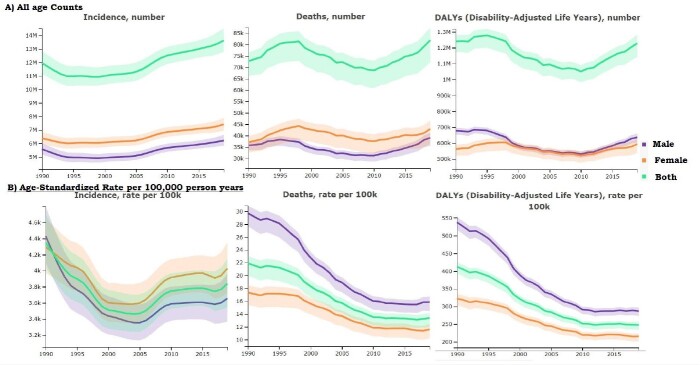

A) All age-counts, Incidence, Death, DALY B) Age-standardized rate Incidence, Death, DALY

Sex-wise distribution of Lower Respiratory Tract Infection, Deaths, per 100,000 across the United States

**Figure:**
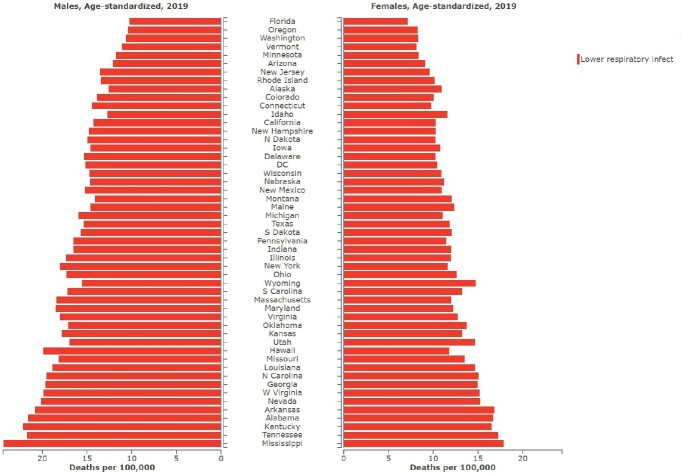

**Conclusion:**

LRIs continue to be a significant burden on the healthcare system in the United States, causing a significant economic impact and affecting vulnerable populations disproportionately. Preventive measures and prompt treatment can help reduce this burden and improve the overall health of the population.

**Disclosures:**

**All Authors**: No reported disclosures

